# O068. A 2 years prospective evaluation study on onabotulinumtoxinA 195 U in chronic migraine

**DOI:** 10.1186/1129-2377-16-S1-A177

**Published:** 2015-09-28

**Authors:** Andrea Negro, Lidia D'Alonzo, Noemi Lala, Paolo Martelletti

**Affiliations:** Department of Clinical and Molecular Medicine, Sapienza University of Rome, Sant'Andrea Hospital, Rome, Italy

## Background

OnabotulinumtoxinA (Botox^®^) is the first and so far the only treatment to receive a specific license for prevention of chronic migraine (CM). In our Headache Clinic the therapy with onabotulinumtoxinA is routinely administered to CM patients on a daily basis since 2001. Preventive treatment with onabotulinumtoxinA was offered to all patients that were 1) adults; 2) fulfilling the ICHD-II criteria for CM with or without analgesic overuse; and 3) with contraindications or lack of efficacy or tolerability to other preventive drugs.

Exclusion criteria were coexistent of neuromuscular disorders, psychiatric diseases considered incompatible with such kind of treatment, pregnancy and breast-feeding.

## Objectives

To prospectively evaluate the variations in terms of headache days, migraine days, acute pain medication intake days through a period of 24 months in comparison to a one-month baseline period before starting the therapy.

## Methods

Among all the patients that from 2011 to 2012 underwent treatment with onabotulinumtoxinA we randomly selected 100 CM patients (F 85 / M 15; mean age 45.4, range 18-75 years; 93% drugs overusers) that were able to fill diaries without any lack of information for a period of 2 years. OnabotulinumtoxinA 195 U was injected in 39 sites combining the PREEMPT “fixed sites/fixed doses” and the “follow the pain” injection paradigm every three months (± one week)[1]. Patients with criteria for medication overuse headache underwent withdrawal and detoxification therapeutic regimen before starting the treatment. Patients were not allowed to continue preventive oral medication during treatment with onabotulinumtoxinA.

## Results

The efficacy results for each timeline are reported in Table 1. The reduction in terms of headache and migraine days, acute medication intake days and HIT-6 score increases strongly from the first injection to the fourth, and remains stable until the last injection at 24 months.

**Table 1:** Efficacy of onabotulinumtoxinA 195 U.

	baseline	3 m	6 m	9 m	12 m	15 m	18 m	21 m	24 m
Headache days	22.2	-8.1	-12	-14.7	-16.5	-16.8	-17.2	-17.7	-18.1
Migraine days	21.6	-7.3	-10.4	-13.1	-15.9	-16.7	-17	-17.5	-17.6
Acute pain medication intake days	21	-7.1	-11.1	-13.8	-15.7	-16	-16.2	-17	-17.3
HIT-6 score	67.9	--	-7	--	-11.1	--	-13.9	--	-19

## Conclusions

Our results support the findings of the PREEMPT study in a large cohort of patients and are representative of the patients observed in a tertiary headache centre.

Written informed consent to publish was obtained from the patient(s).
